# Experiencing a first food allergic reaction: a survey of parent and caregiver perspectives

**DOI:** 10.1186/1710-1492-7-S2-A6

**Published:** 2011-11-14

**Authors:** Zainab Abdurrahman, Monika Kastner, Cory Wurman, Laurie Harada, Laura Bantock, Susan Waserman

**Affiliations:** 1 Division of Clinical Immunology and Allergy, McMaster University, Hamilton, Ontario, Canada; 2 Li Ka Shing Knowledge Institute of St. Michael’s Hospital, Toronto, Ontario, Canada; 3 Dept of Health Science, UWO, London, Ontario, Canada; 4 Anaphylaxis Canada, Toronto, Ontario, Canada

## Background

Inadequate knowledge of food allergy and anaphylaxis has been identified by caregivers as an important barrier to coping, and a potential cause of fear and anxiety. This is especially true for those newly diagnosed with food allergy.

## Objective

The objectives of this research study were to better understand the experiences of caregivers of children with newly diagnosed food allergy (first allergic reaction within the last 12 months), and to identify the information gaps between what caregivers received at diagnosis and what they perceived they needed.

## Methods

An online survey was administered to members of Anaphylaxis Canada (a patient support group consisting of approximately 12,000 members). The sampling strategy included an email invitation, and posting of a URL link on the organization’s website.

## Results

Of 293 respondents, 208 were eligible (newly diagnosed), and 184 consented. 83.5% of respondents reported being anxious to extremely anxious at first diagnosis, and only 38% stated that they had received enough information. Identified gaps included education on food allergy, anaphylaxis management, how to use epinephrine auto-injectors, and coping strategies. Actions taken by families in response to the diagnosis included avoidance of eating out at restaurants (85%), restriction of their child’s activities with other children (61%), limitation of travel (49%), termination of their job (11%), and a reduction in work hours (13%).

## Conclusions

Survey findings will be supplemented by a follow-up qualitative study to better understand gaps. These findings will then inform the development of educational strategies for patients newly diagnosed with food allergy.

